# Exploring cross-regional and cross-variable transferability of a ResNet-based super-resolution method for the ERA5 data

**DOI:** 10.1038/s41598-026-41002-7

**Published:** 2026-02-25

**Authors:** Zijun Li, Hoiio Kong, Chanseng Wong, Chilam Ao, Yu Du

**Affiliations:** 1 Faculty of Data Science, City University of Macau, Macau, 999078 China; 2 Keang Peng School, Macau, 999078 China; 3 School of Atmospheric Sciences, Sun Yat-Sen University and Southern Marine Science and Engineering Guangdong Laboratory (Zhuhai), Zhuhai, 519082 China

**Keywords:** Transfer learning, Super-resolution, ResNet, ERA5 data, Climate sciences, Environmental sciences, Mathematics and computing

## Abstract

With the development of artificial intelligence, diverse datasets can be assisted in refined operations AI. In recent years, AI has been applied to meteorological data forecasting. However, using AI presents challenges such as long training times and high computational costs. Applying similar meteorological data models across different regions to reduce repetitive training costs remains a significant issue to address. This study explores the transfer learning capabilities of a super-resolution (SR) reconstruction model using 2-meter temperature data from SouthChina. The ResNet, integrated with sub-pixel convolution modules, effectively captures data features. By leveraging similar temperature data across different regions, the model’s SR reconstruction performance is evaluated. Experiments compare the model’s transfer learning abilities across various regions. Additionally, given the correlation of meteorological features within the same region, the study attempts to reconstruct other meteorological data (e.g. wind speed, atmospheric pressure, etc.) with SR. Both 2x and 4x SR experiments for the two tasks yield favorable results. Compared to traditional interpolation methods, the transfer learning-based neural network model produces more accurate outcomes. The findings indicate that neural network models possess strong transfer learning capabilities, which are highly significant for climate research and related applications and confirm the feasibility of transfer learning in meteorological data.

Since the 21st century entered the era driven by big data, numerous industries have witnessed rapid development propelled by more refined and extensive datasets^1,2^. In recent years, growing attention to meteorology and environmental protection has spurred demands for greater accuracy and speed in meteorological forecasting^3,4^. Leveraging big data technologies to enhance the quality of forecasts has become a priority. Temperature data, being crucial for meteorological research, requires particular attention regarding its quality. Correspondingly, temperature data collection has emerged as a key task within meteorological agencies.

To improve the quality of temperature data collection, a variety of distinct data collection methods have been developed. These include remote sensing technologies (e.g., remote sensing and GPS)^5^, which can collect data across large areas simultaneously but have low and fixed sampling frequencies. The unmanned aerial vehicles (UAVs) are used for data collection in uninhabited areas such as high altitudes and mountainous regions^6^. In urban areas, Automatic Weather Stations (AWS) or surface - based observatories are deployed, and temperature data is collected by meteorological professionals^7^. Despite having various temperature data collection methods, meteorological agencies face difficulties due to insufficiently refined and unevenly distributed data collection across regions and countries because of economic and technical disparities. Under traditional methods, researchers often use data assimilation to fill in or perform super - resolution (SR) operations on missing or incomplete temperature data^8,9^. However, this method consumes significant computational resources and time. To reduce computational and temporal costs, researchers have begun to leverage artificial-intelligence technologies to assist in data completion^10^. With the advancement of technology, some researchers have attempted to use it to achieve SR operations on temperature data. Compared with data assimilation, artificial intelligence is less time-consuming. Unfortunately, a single AI model must be trained for a specific region and dataset in practice, which means different prediction models need to be separately developed for different cases. This leads to low model utilization and duplication of effort. Moreover, obtaining datasets for training various models is also a tough task for researchers.

To address the issues of repetitive training and difficulty in obtaining training data, researchers have begun to consider transfer learning using similar models. By leveraging the deep feature extraction capabilities of neural networks, pre-trained models can be applied to similar tasks. This approach aims to reduce the amount of data required and minimize training time^11^. To circumvent the excessive repetitiveness of machine learning methods in practical temperature data SR tasks and enhance efficiency, researchers have sought solutions. However, the reliability of model generalizability remains a concern in meteorological data SR research^12–15^.

Our study offers a more efficient way to apply AI in meteorological data-related fields through model transfer learning. Using a ResNet-based SR model, we explore model’s transfer learning ability in 2-meter-temperature (2 m-T) data SR tasks. The model takes low-resolution (LR) data of different sizes as input and outputs high-resolution (HR) data. This paper proposes to adapt a SR model, initially trained on a region-specific dataset, to diverse meteorological datasets for the generation of HR meteorological data. The transferability of the model will be assessed based on the experimental outcomes. The investigation into the model’s transfer learning capability is structured into two components: first, evaluating the super-resolution performance on temperature data across different geographical regions; and second, examining the super-resolution results for various meteorological variables within the same region, including Mean Sea level pressure (MSLP), 10-meter-U wind component (10 m-U), 10-meter-V wind component (10 m-V) and 2-meter dewpoint temperature (2 m-D). In the field of meteorology, temperature, wind, and atmospheric pressure are factors of primary concern. Efficient and convenient access to high-precision meteorological data is crucial for forecasting operations and subsequent research^16^. Comparing the model with traditional interpolation methods shows that transfer learning is feasible for SR tasks of temperature and related meteorological data. In summary, the contributions of this paper are as follows:

1. Conducted SR reconstruction of 2 m-T data across different regions using transfer learning and assessed its effectiveness. Compared with traditional interpolation methods, the feasibility of transfer learning was proven.
2. Used transfer learning to perform super - resolution reconstruction of different meteorological data within the same region and evaluated its effectiveness.
3. Analyzed the factors affecting transfer learning outcomes and identified regions more suitable for it.

The structure of the remainder of this paper is as follows: Sect. “Related works” reviews the relevant research work. Section “Method” presents the datasets used and the delineation of transfer - learning regions. Section “Experiments and results” outlines the ResNet model and the super - resolution reconstruction method. Section “Discussion” reports the experimental results and discusses the feasibility and effectiveness of the proposed method. Section “Conclusions” concludes the study and discusses potential directions for future research.

## Related works

### Deep learning

With the advancement of deep learning, neural networks have shown remarkable potential in SR^17–19^. They perform feature extraction to nonlinearly map the feature space of LR data to that of HR data, filling in missing information and enhancing image accuracy.

Similarly, some researchers have applied neural networks to the SR of meteorological data. Sha enhanced the standard U-Net by appending HR reconstruction branch^20^. This design enables the model to perform unsupervised fine-tuning on incomplete target-region data and to adapt to local characteristics without requiring labelled samples. In 2021, Medina proposed a framework that integrates bias adjustment with a convolutional neural network (CNN)^21^. By applying temporal Bias Adjustment and standardization to the outputs of the EC-Earth model, their approach preserved spatial coherence during the transition from gridded fields to discrete station data. Capitalizing on the representational strength of the Transformer, Wang pioneered its application to the SR of temperature dataset^22^. A multi-scale feature-fusion module allows the network to perform simultaneous SR and bias adjustment, while a terrain features block enhances the model’s capacity to capture fine-scale structures over complex terrain.

It integrates a Channel-Spatial Attention Mechanism into traditional residual modules. For near-surface climate data research, Jiang proposed a method based on the Fourier Neural Operator (FNO)^23^. It incorporates the Clausius-Clapeyron equation into spatial transformation to describe the constraints between meteorological quantities. In addition to spatial physical constraints, Wang et al. considered temporal correlations and proposed the T-INRI model^24^. Time data is embedded in temperature data expressed as a continuous function through a time-aware implicit neural representation. The model weights four LR data points near the prediction point, addressing spatial discontinuities. Chen proposed the CS-Diffusion method, a dual-framework model^25^. The first layer is a Pre-SR Network for preliminary data processing using reference images. The second layer is a denoising layer by U-net. It incorporates the Cross-Scale Reference Image Attention Mechanism (CSRIAM) to address feature loss during downsampling. Table 1 summarizes the related deep learning research.

**Table 1:** Summary of deep learning research in SR.

Model	Technological features	Limited
Sha-UNet^20^	Appending HR branch	Exhibits excessive smoothing
Medina ^21^	Bias Adjustment to maintain data spatial consistency	No correlation between discrete points
SwinIR & Uformer^22^	Multi-scale feature-fusion & terrain features block	Time-independent dependency
FNO^23^	Physically constrained	Poor at land-ocean boundaries
T_INRI^24^	Time-aware embedding & multi-neighbor weighting	Time data effective only at small scales
CS-Diffusion^25^	Pre-SR & CSRIAM	Time-consuming

### Transfer learning

Much research on transfer learning exists, but Weiss formally proposed the concept^11^. He defined it as using labeled data or knowledge structures from related tasks to optimize the training of a target task.

Zhang et al. first employed SIFT feature matching to filter out suitable images, increasing training data^26^. Bicubic interpolation was used in shallow layers. Although interpolation mitigated overfitting, it introduced a problem: the fixed bicubic interpolation is untrainable and non-adaptive, restricting the model’s flexibility. Gil-Gamboa using Deep Feed-Forward Neural Networks (DFFNN)^27^, transfer learning for water usage data by electricity consumption data. By analyzing the seasonal fluctuations, K-means clustering identifies water usage patterns (industrial or residential). However, the model is difficult for transferring learning to outlier.

Transfer learning for temperature data was initially studied for indoors. Bellagarda proposed a two-stage transfer learning framework^28^, pre-training models on Building Information Modeling (BIM) EnergyPlus simulation data and fine-tuning them with real data. Focusing on near-surface temperature, Wang proposed TranSAT^29^, which integrates U-Net and DNN. The study used multiple aligned spatial-scale heterogeneous datasets (e.g., satellite remote sensing, terrain) to increase data dimensionality. However, excessive data volume leads to training time becoming rather long. Table 2 summarizes the related transfer learning research.

**Table 2:** Summary of transfer learning research in meteorological data.

Model	Technological features	Limited
SIFI-CNN^26^	SIFI matching	Interpolation is untrainable
DFFNN^27^	K-means preprocessing	Poor at outlier
Bellagarda^28^	2-stage transfer learning	High dataset requirements
TranSAT^29^	DNN realizes U-net framework	Excessive data increases training consumption

### Data

This study uses ResNet-Subpixel^30^ and data for pre-training. The data is from the European Centre for Medium - Range Weather Forecasts (ECMWF) Re - Analysis v5 (ERA5) dataset. ERA5 offers high-resolution atmospheric, land, and ocean variables^31^. It covers hourly instant data since 1950 and is a key dataset for meteorological and deep learning research^32–35^.

**Fig. 1:**
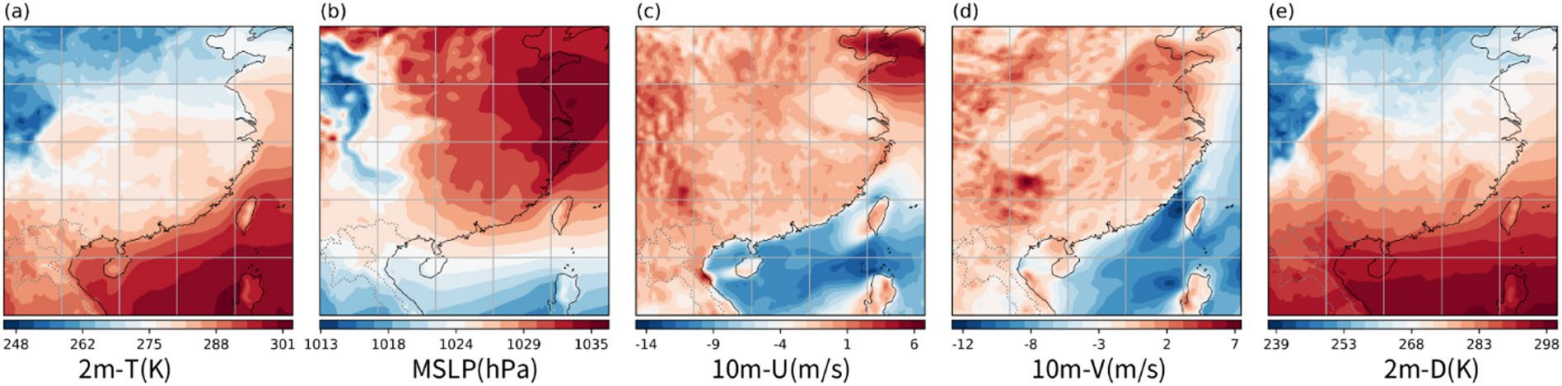
(**a**) 2 m-T, (**b**) MSLP, (**c**) 10 m-U, (**d**) 10 m-V and (**e**) 2 m-D at 0000 UTC 1 January 2020. The presented data is prepared by torch v.2.8.0 + cu126 https://pytorch.org/ and plotted by Matplotlib v.3.10.0 https://matplotlib.org/.

Figure 1 shows the data variables used in this study^36^: (a) 2 m-T, (b) MSLP, (c) 10 m-U, (d) 10 m-V and (e) 2 m-D. The 2 m-T variable includes pre-training and geographical transfer learning data, while the others represent different meteorological data for transfer learning. All are derived from single level ERA5 hourly data with a 0.25°×0.25° resolution, equivalent to a 25 km interval.

**Fig. 2:**
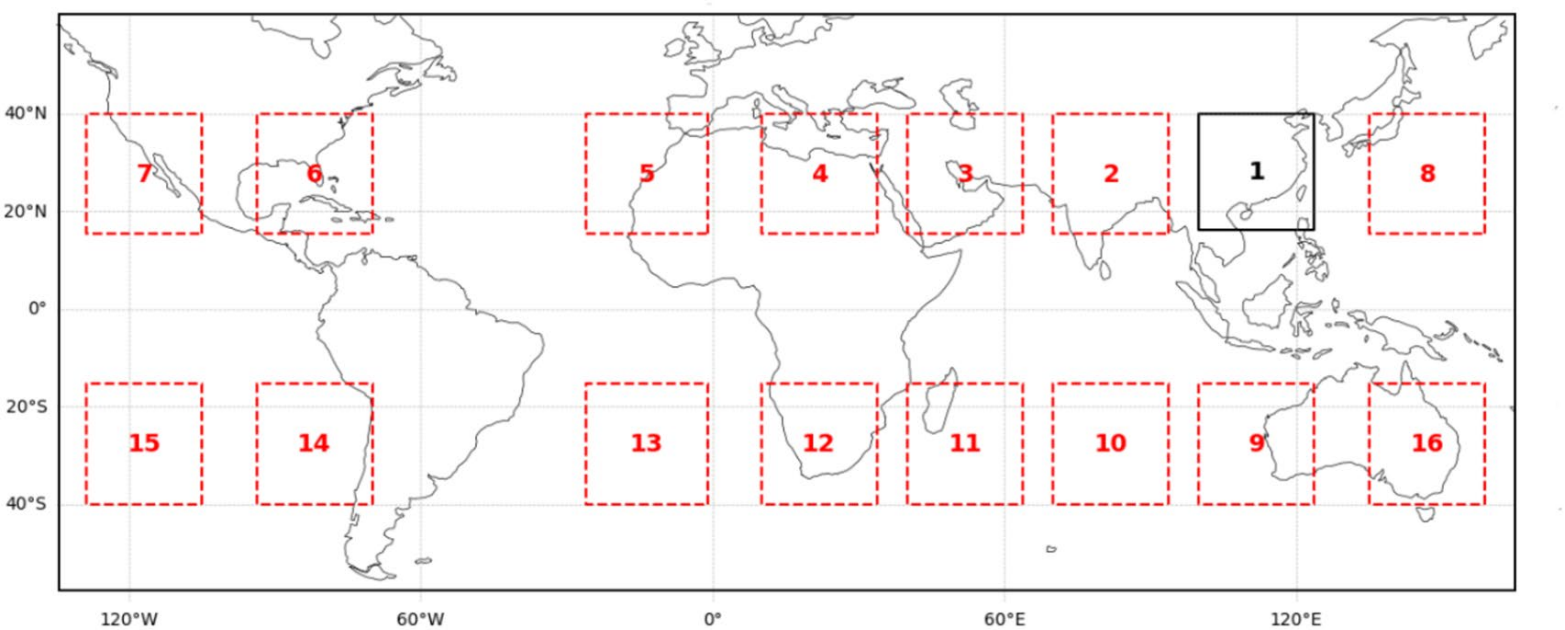
Map of the 16 study boxes. Data is prepared by cartopy v. 0.25.0 https://scitools.org.uk/cartopy/docs/latest/ and plotted by Matplotlib v.3.10.0 https://matplotlib.org/.

We delineate 16 research regions, termed Boxes. Box1, a 23.75 × 23.75° square area, serves as the pre-training data region. Boxes 2–16, for transfer learning, match Box1 in size. The study uses data from January 1 to December 31, 2020, with each Box containing about 17,520 samples. The geographic locations of the Boxes are shown in Fig. 2, with minimum, maximum, and mean annual data (Min, Max, Mean) in Table 3. For original data, we perform downsampling to obtain LR data as model input. In the experiment, we use 2 scales (2× and 4×) to generate LR datasets. We design window sizes (2 × 2 or 4 × 4 pixels) based on the downsampling scale and calculate the data point values within each window. To ensure alignment between the downsampled and original data, we discard incomplete border data points. Overall, we generate LR datasets for initial model pre-training. Subsequently, the pre-trained model is transferred to similar datasets. By performing SR on the LR data and comparing it with the original data, we verify the model’s transfer learning ability.

**Table 3:** Location, minimum 2 m-T, maximum 2 m-T, and mean 2 m-T of the 16 boxes.

Box	Location	Min (T /K)	Max (T/K)	Mean (T/K)
1	100-123.75° E 16.25-40° N	234.74	318.12	291.49
2	70-93.75° E 16.25-40° N	225.96	321.94	287.96
3	40-63.75° E 16.25-40° N	237.44	326.03	295.95
4	10-33.75° E 16.25-40° N	250.19	320.76	295.68
5	155.25–179° W 16.25-40° N	259.42	322.60	295.61
6	86.25–110° W 16.25-40° N	251.29	318.22	295.71
7	106.25–130° W 16.25-40° N	240.84	323.97	292.5
8	135-158.75° E 16.25-40° N	252.31	314.69	295.82
9	100-123.75° E 16.25-40° S	272.08	320.96	293.81
10	70-93.75° E 16.25-40° S	277.53	303.28	293.01
11	40-63.75° E 16.25-40° S	274.58	315.71	294.16
12	10-33.75° E 16.25-40° S	257.69	318.58	292.76
13	155.25–179° W 16.25-40° S	276.44	301.03	292.91
14	86.25–110° W 16.25-40° S	248.00	310.69	289.99
15	106.25–130° W 16.25-40° S	278.90	300.89	293.09
16	135-158.75° E 16.25-40° S	264.19	320.64	294.27

## Method

As previously mentioned, this study utilizes ResNet-SubPixel^30^ as the pre-trained model and maintains consistency in its application throughout the subsequent research.

### ResNet-subpixel

**Fig. 3:**
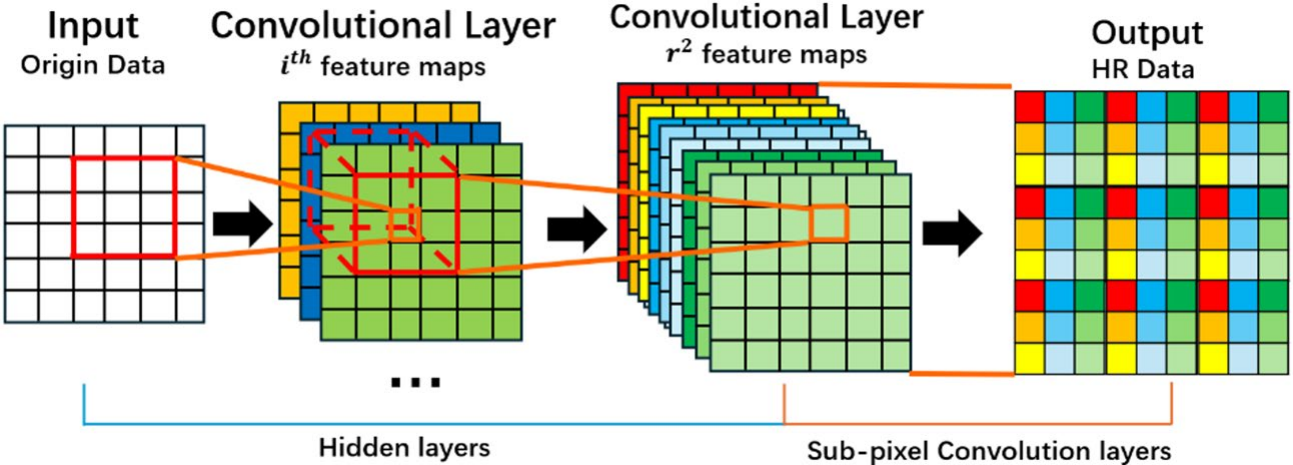
Sub-pixel convolution process. The image is produced by the Photoshop v.2024 (25.0) https://www.adobe.com/products/photoshop.html.

ResNet-Subpixel is a model that combines Sub-Pixel Convolution with residual blocks. Unlike traditional SR models that use upsampling computations for predictions, it rearranges the feature matrices in the model’s hidden layers to obtain high-resolution data. As shown in Fig. 3, the model sets the feature channels to a size of $\:{r}^{2}$. During upsampling, the feature matrices fill these channels, and after periodic shuffling, HR data of size $\:r\times\:W$ and $\:r\times\:H$ are obtained^37^, and the expression is as shown in Eq. (1).

1 $$\begin{array}{*{20}{c}} {N*\left( {C*r*r} \right)*W*H \Rightarrow N*C*\left( {W*r} \right)*\left( {H*r} \right)~} \end{array}$$

where, $\:N\:$denotes the Batch Size; $\:C$ denotes the number of channels; $\:W,\:H$ are the dimensions of the LR data; $\:r$ denote upscale factor, which determines the SR ratio. During the training process, the model adjusts the weights of each channel. This makes the upsampling process a non-linear mapping rather than a linear one. As a result, it reduces the influence of researcher subjectivity in the SR process.

**Fig. 4:**
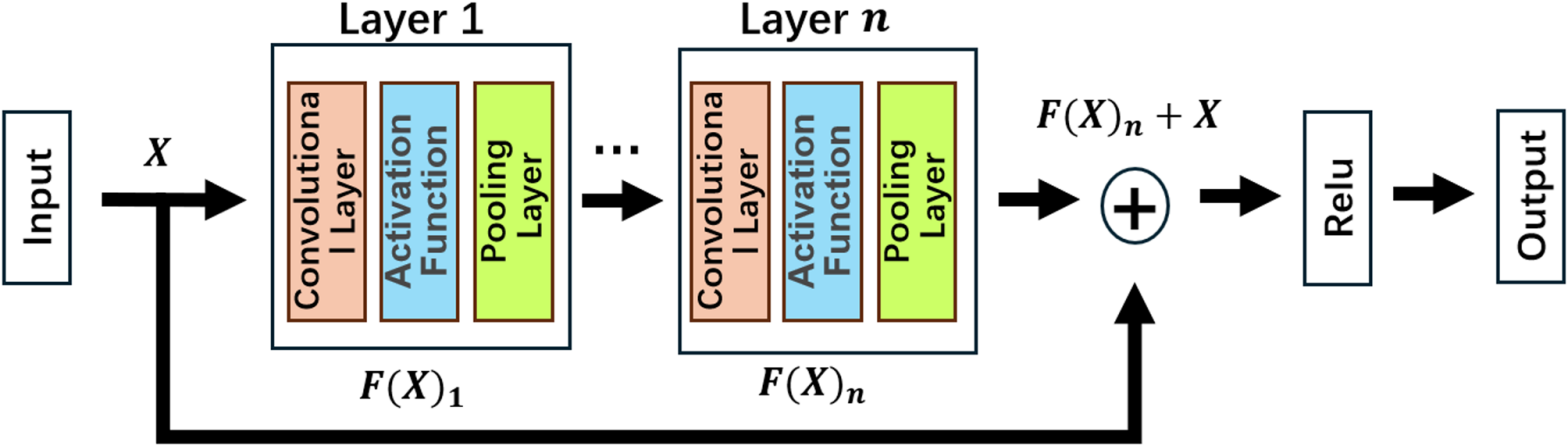
Residual networks. The image is produced by the Photoshop v.2024 (25.0) https://www.adobe.com/products/photoshop.html.

The use of convolutional kernels is proposed for feature extraction, enabling the emergence and rapid development of CNNs^38^. This has led to more complex and efficient models in fields such as images and text^39,40^. However, overly complex models increase the risk of overfitting^41^. ResNet reduces model complexity while maintaining performance through skip connections^42^. As shown in Fig. 4, residual blocks connect layers of different depths, preventing the amplification of improper weight parameters during training and avoiding gradient vanishing. The model training process is represented by Eq. (2).

2 $$\begin{array}{*{20}{c}} {{f^n}\left( {I;{W_1},{b_1}, \ldots ,{\mathrm{~}}{W_{\mathrm{n}}},{b_{\mathrm{n}}}} \right)=\sigma \left( {{W_n}{\mathrm{*}}{f^{n - 1}}\left( {I;{W_1},{b_1},{\mathrm{~}} \ldots ,{\mathrm{~}}{W_{{\mathrm{n}} - 1}},{b_{{\mathrm{n}} - 1}}} \right)+{b_n}} \right)+F\left( I \right)~} \end{array}$$

where $\:{f}^{n}$ denotes the convolution operation of the $\:n$-th layer; $\:{W}_{i}$ and $\:{b}_{i}$ represent the weight and bias parameters of the $\:n$-th layer, respectively; $\:I$ denotes the input data; and $\:\sigma\:$ is the activation function; $\:F$ denotes a residual block. During model training, the primary objective is to establish an identity mapping between the input ($\:I$) and the desired output ($\:f\left(I\right)$), as $\:f\left(I\right)=I$. Unfortunately, directly identifying this mapping in complex problems is challenging and has led to the development of more intricate models. Through the introduction of residual blocks, this problem is translated into $\:f\left(I\right)=I+F\left(I\right)$, with the identity mapping serving as an integral component of the network. ResNet focuses on solving $\:F\left(I\right)=f\left(I\right)-I$, when $\:F\left(I\right)=0$, training is considered complete^43^. By not directly solving the identity mapping, training difficulty and complexity are significantly reduced.

**Fig. 5:**
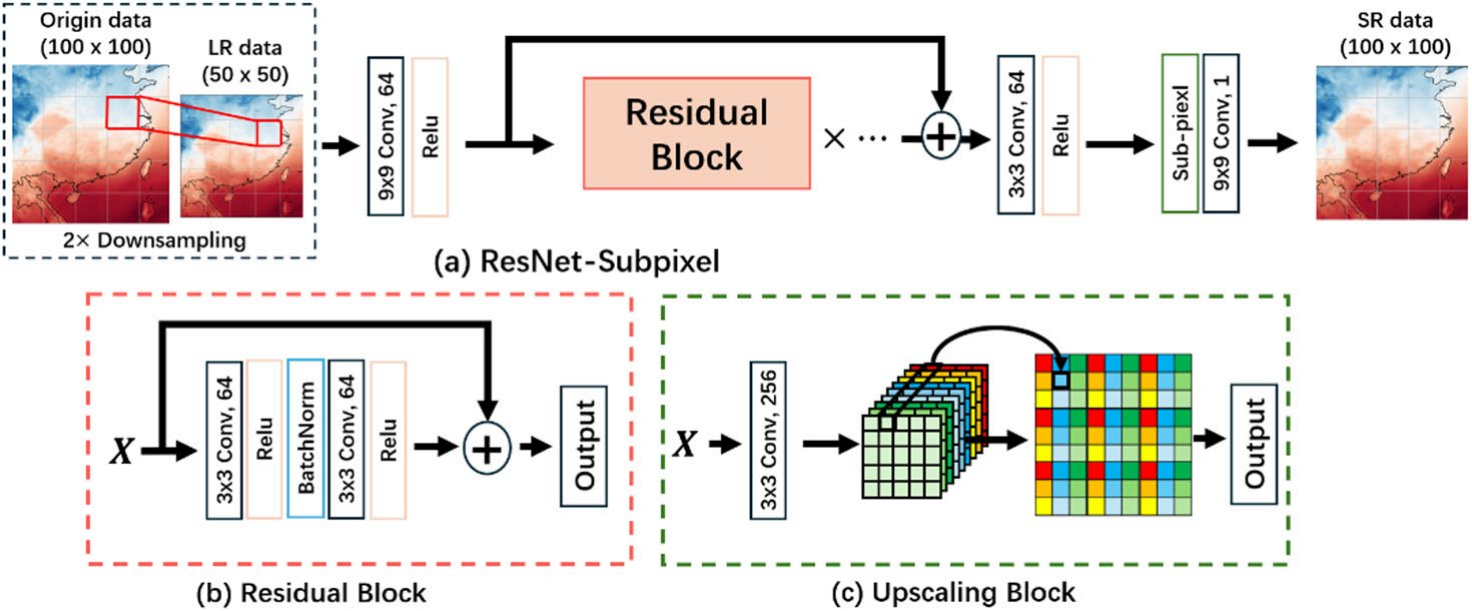
(**a**) ResNet-Subpixel (2×) structure and experimental flow, containing some residual blocks. (**b**) Residual block containing 2 convolutional layers, 2 BatchNorm layers, and an activation function Relu. (**c**) Upscaling Block. The image is produced by the Photoshop v.2024 (25.0) https://www.adobe.com/products/photoshop.html.

We pre-train ResNet-Subpixel using the data within Box1. The training dataset is 2 m-T data, which consists of data from January 1, 2023 to December 31, 2023. During the experiment, we first downsample the original data to generate LR data. The data is then fed into the model, where feature extraction is performed using residual blocks. Subsequently, the Sub-pixel layer is employed to carry out the SR operation. For the specific process, refer to Fig. 5.

**Fig. 6:**
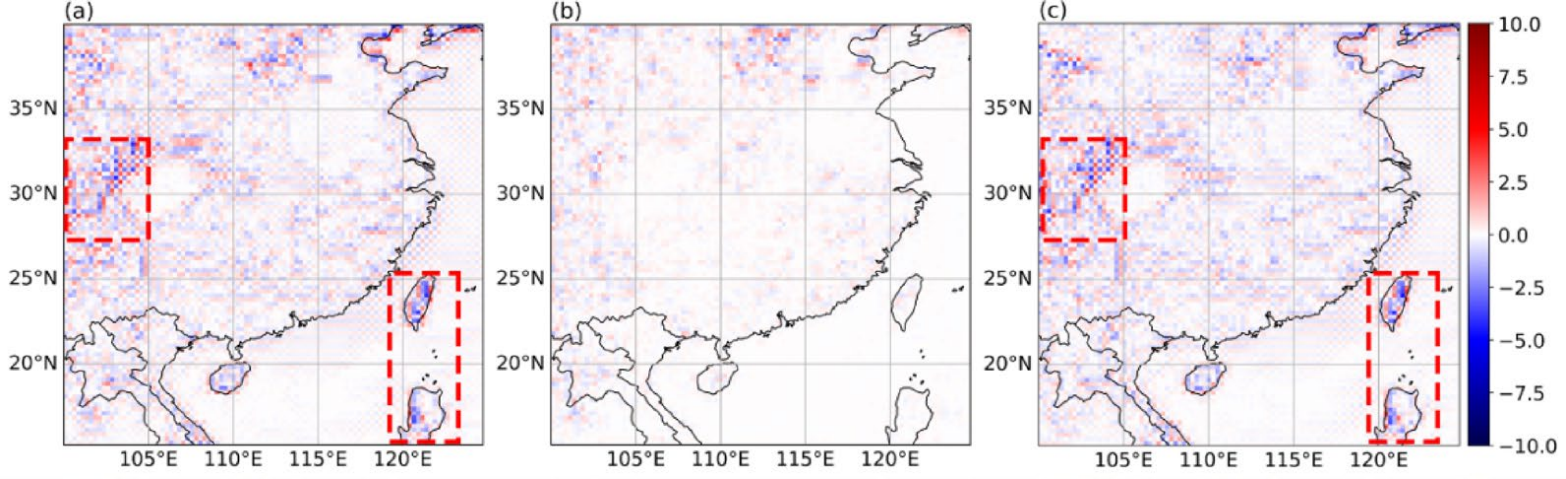
(**a**) Comparison between real data and LR data, (**b**) comparison between real data and SR data, and (**c**) comparison between SR data and LR data^28^. The presented data is prepared by torch v.2.8.0 + cu126 https://pytorch.org/ and plotted by Matplotlib v.3.10.0 https://matplotlib.org/.

After pre-training, the model has effectively learned the features of the 2 m-T data within box1 and demonstrates excellent SR performance in the region. The SR of sample data is shown in Fig. 6, it can be observed that the differences between the SR data and the true data (Fig. 6b) are minimal, with deviations primarily occurring in inland regions. Comparing Fig. 6a with Fig. 6c, the overall patterns are quite similar, with the main deviation areas and directions being consistent (areas designated by the red dotted lines in Fig. 6a and c).

### Model evaluation

**Fig. 7:**
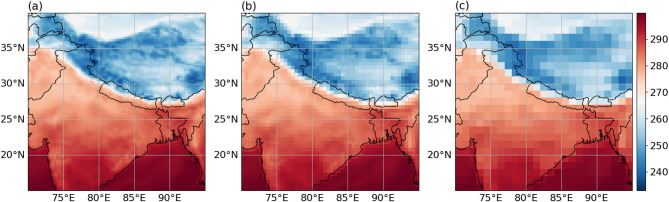
2 m-T in the region of box 2 at 0000 UTC 1 January 2020 with (**a**) original resolution of 0.25°×0.25°, (**b**) low-resolution dataset of 0.5°×0.5°, and (**c**) low-resolution dataset of 1°×1°. The presented data is prepared by torch v.2.8.0 + cu126 https://pytorch.org/ and plotted by Matplotlib v.3.10.0 https://matplotlib.org/.

To evaluate the model’s performance, we analyze its SR reconstruction of meteorological data with a spatial resolution of 0.25°×0.25° within each Boxes, conducting multiple experiments. First, we assess the model’s reconstruction ability across different SR scales. We downsample the original data at 2x and 4x to produce multiple sets of LR data with spatial resolutions of 0.5°×0.5° and 1°×1°. Figure 7 presents a single moment data sample from Box2. Second, for each LR data, we perform two tasks: (1) evaluating the SR model performance at a single moment and (2) assessing the SR model performance over an average moment (for the test dataset average). Additionally, we compare our model with conventional machine learning methods to conduct a comprehensive analysis of its performance.

The assessment of the model performance was carried out from multidimensional metrics containing Root Mean Square Error ($\:RMSE$), Mean Absolute Error ($\:MAE$), Mean Absolute Percentage Error ($\:MAPE$), correlation coefficient ($\:Corr$), Peak Signal-to-Noise Ratio ($\:PSNR$), and structural similarity ($\:SSIM$). The formula for the evaluation indicators is as follows:

3 $$\begin{array}{*{20}{c}} {RMSE\left( {x,y} \right)=\sqrt {\frac{{\mathop \sum \nolimits_{{j=1}}^{P} \mathop \sum \nolimits_{{i=1}}^{N} {{\left( {x_{i}^{j} - y_{i}^{j}} \right)}^2}}}{{N \cdot P}}} } \end{array}$$

4 $$\begin{array}{*{20}{c}} {MAE\left( {x,y} \right)=\frac{{\mathop \sum \nolimits_{{j=1}}^{P} \mathop \sum \nolimits_{{i=1}}^{N} \left| {x_{i}^{j} - y_{i}^{j}} \right|}}{{N \cdot P}}} \end{array}$$

5 $$\begin{array}{*{20}{c}} {MAPE\left( {x,y} \right)=\frac{{100}}{{N \cdot P}}\mathop \sum \limits_{{j=1}}^{P} \mathop \sum \limits_{{i=1}}^{N} \left| {\frac{{x_{i}^{j} - y_{i}^{j}}}{{x_{i}^{j}}}} \right|} \end{array}$$

6 $$\begin{array}{*{20}{c}} {Corr\left( {x,y} \right)=\frac{{\mathop \sum \nolimits_{{j=1}}^{P} \mathop \sum \nolimits_{{i=1}}^{N} \left( {x_{i}^{j} - {{\bar {x}}^j}} \right)\left( {y_{i}^{j} - {{\bar {y}}^j}} \right)}}{{\sqrt {\mathop \sum \nolimits_{{j=1}}^{P} \mathop \sum \nolimits_{{i=1}}^{N} {{\left( {x_{i}^{j} - {{\bar {x}}^j}} \right)}^2}} \sqrt {\mathop \sum \nolimits_{{j=1}}^{P} \mathop \sum \nolimits_{i}^{N} {{\left( {y_{i}^{j} - {{\bar {y}}^j}} \right)}^2}} }}} \end{array}$$

7 $$\begin{array}{*{20}{c}} {PSNR\left( {x,y} \right)=10 \cdot {{\log }_{10}}\left( {\frac{{MA{X^2}}}{{RMSE{{\left( {x,y} \right)}^2}}}} \right)} \end{array}$$

8 $$\begin{array}{*{20}{c}} {SSIM\left( {x,y} \right)=\frac{{\left( {2{\mu _x}{\mu _y}+{C_1}} \right)\left( {2{\sigma _{xy}}+{C_2}} \right)}}{{\left( {\mu _{x}^{2}+\mu _{y}^{2}+{C_1}} \right)\left( {\sigma _{x}^{2}+\sigma _{y}^{2}+{C_2}} \right)}}} \end{array}$$

where $\:P$ is the total number of the samples; $\:N$ is the total number of pixels in one sample; $\:{x}_{i}^{j}$ and $\:{y}_{i}^{j}$ denote the $\:i$-th pixel at the $\:j$-th moment in the original results and the SR data, respectively; $\:{\stackrel{-}{x}}^{j}$ and $\:{\stackrel{-}{y}}^{j}$ are the mean values of $\:{x}^{j}$ and $\:{y}^{j}$ at the $\:j$-th moment, respectively.

$\:RMSE,$ $\:MAE$, and $\:MAPE$ are common machine learning metrics that intuitively show the differences between model outputs and real data^44–46^. $\:RMSE$ measures the average difference, $\:MAE$ assesses the absolute error, and $\:MAPE$ evaluates the relative percentage error. These three metrics effectively help in understanding the differences between SR data and real data, as well as analyzing the magnitude and bias. However, these metrics are sensitive to outliers and cannot accurately reflect overall correlations. Therefore, we introduce three additional metrics: $\:Corr$, $\:PSNR$ and $\:SSIM$. $\:Corr$ does not measure the magnitude of differences between two variables but rather assesses the strength of their linear relationship using the Pearson Correlation Coefficient^47^. $\:PSNR$ is used to evaluate the quality of images or videos by describing the signal-noise ratio^48^. In machine learning, it measures data distortion, where MAX is the maximum possible value of the data. $\:SSIM$ measures the similarity of structural information by assessing the distortion between two images based on luminance, contrast, and structure^49^. Where $\:{\mu\:}_{x}$ and $\:{\mu\:}_{y}$ represent the average brightness of images $\:x$ and $\:y$; $\:{\sigma\:}_{x}\:\mathrm{a}\mathrm{n}\mathrm{d}\:{\sigma\:}_{y}$ denote the standard deviation (contrast), respectively; $\:{\sigma\:}_{xy}$ denotes the covariance between $\:x$ and $\:y$ (structural).

### Hardware and software packages

All experiments in this study were conducted on Google Colaboratory, with deep-learning models trained and evaluated on Tesla T4 GPUs.

To facilitate efficient model pre-training and transfer learning, we leveraged various Python libraries. Xarray (v.2025.3.1) and OS were employed to read ERA5 data for the experiments, the glob was employed to locate files and manage data paths efficiently. To visualize model performance intuitively, we employed Cartopy (v.0.25.0) to obtain geographical information and generated spatial plots of the results. NumPy (v. 2.0.2) serves as the foundational package for numerical computing, supporting a wide range of complex operations. Torch module (v.2.8.0 + cu126), which is a mature and efficient machine-learning library, was utilized for model design and training. We additionally leveraged Scikit-learn (v.1.6.1) for splitting the training set, computing loss functions, and other auxiliary tasks to support model training. SCV (v.1.0) systematically logs and arches all experimental outputs, furnishing a comprehensive data repository for subsequent analytical validation. RegularGridInterpolator, a subclass within SciPy (v.1.16.1), was employed to perform the interpolation operations required for generating LR data. Matplotlib module (v.3.10.0) was utilized to plot experimental results on maps.

## Experiments and results

### Regional transfer

**Fig. 8:**
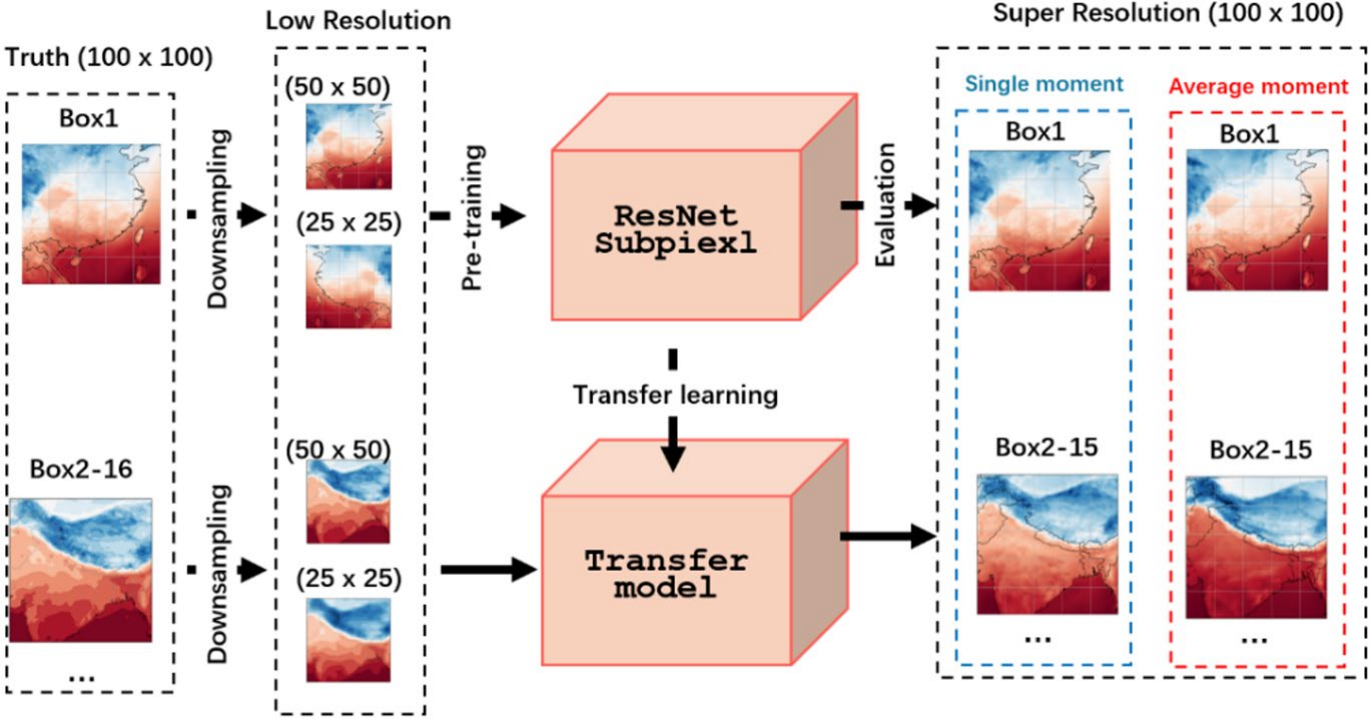
Flow of regional transfer learning experiment. The image is produced by the Photoshop v.2024 (25.0) https://www.adobe.com/products/photoshop.html.

To study the model’s SR performance across different regions, we designed an experiment, as shown in Fig. 8. Box1 as the pre-training region, where the original data (0.25°×0.25°) is downsampling to obtain LR data (0.5°×0.5° and 1°×1°), which are employed to pre-training the ResNet-Subpixel model. For transfer learning, the same processing procedures are applied to the data in Box2-15. Then the SR performance of each box will be evaluated.

**Fig. 9:**
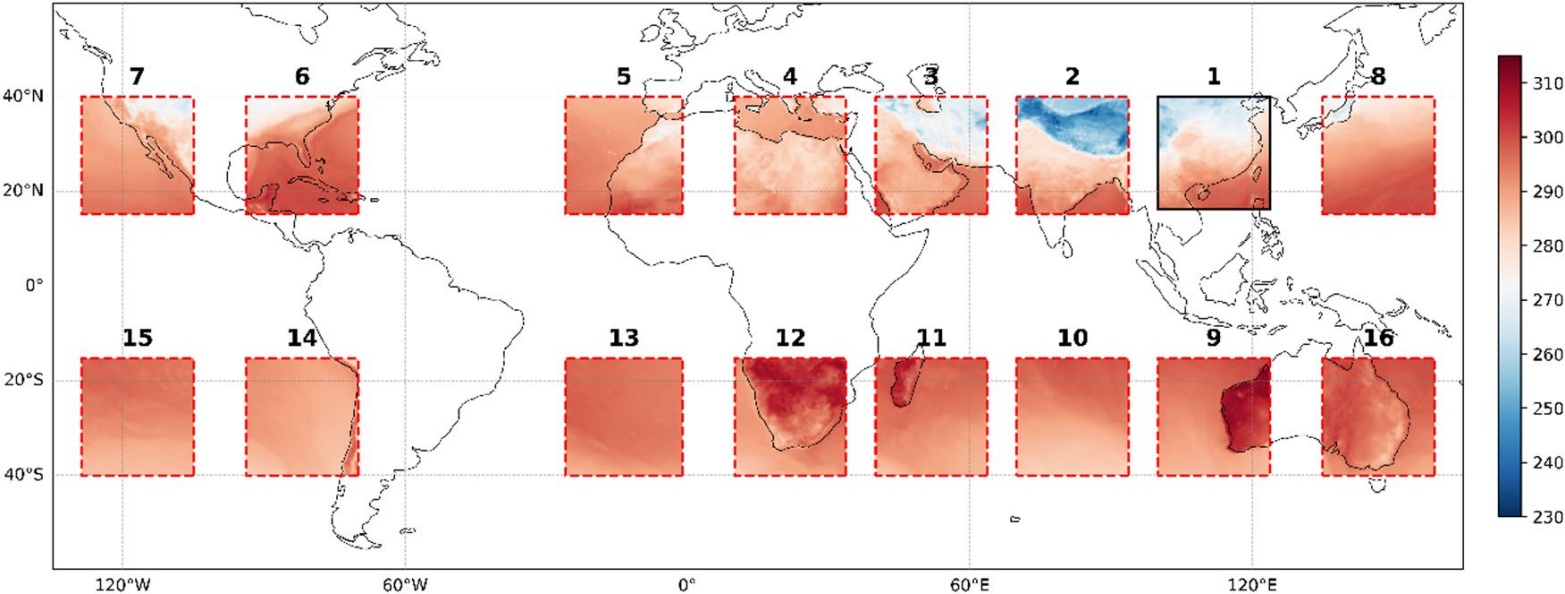
SR results for the single moment across 16 boxes at a resolution of 0.5° × 0.5° at 2300 UTC 31 December 2020. The presented data is prepared by torch v.2.8.0 + cu126 https://pytorch.org/ and plotted by Matplotlib v.3.10.0 https://matplotlib.org/.

**Fig. 10:**
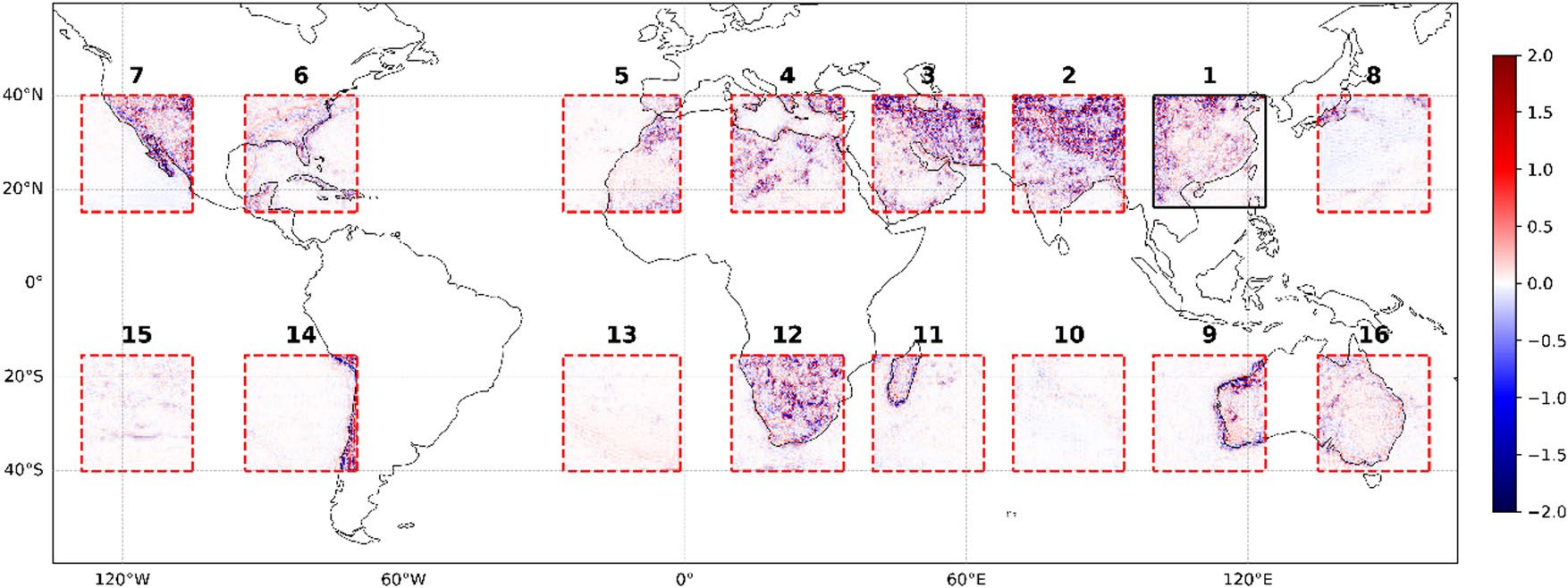
Comparison between real data and SR data for the single moment across 16 boxes at a resolution of 0.5° × 0.5°at 2300 UTC 31 December 2020. The presented data is prepared by torch v.2.8.0 + cu126 https://pytorch.org/ and plotted by Matplotlib v.3.10.0 https://matplotlib.org/.

Figures 9 and 10 show the SR results of 2 m-T data at 2× downsampling for a single moment, and the difference between SR data and real data. The model takes 50 × 50 LR gridded data as input and outputs 100 × 100 SR gridded data. During transfer learning, the model effectively captures features within the Box. Comparison shows that in regional transfer, oceanic regions better SR results than land one. Figure 10 indicates that Boxes 10, 13, and 15, which cover only oceanic regions, have the best performance. Boxes 8, 11, and 14, with mostly oceanic, are the second-best. Other boxes with poorer results contain large land regions. Notably, Box 2, almost entirely covering land, has the worst results, confirming that marine regions are more suitable for transfer learning in this context. The results are primarily attributed to the more uniform and stable environment of oceanic compared to land. As shown on Fig. 9, in oceanic regions like Box15, temperatures are relatively homogeneous. Conversely, land regions like Box2 has complex terrain and highly variable weather, making it difficult for models to complete transfer learning. These factors make transfer learning more challenging over land.

**Fig. 11:**
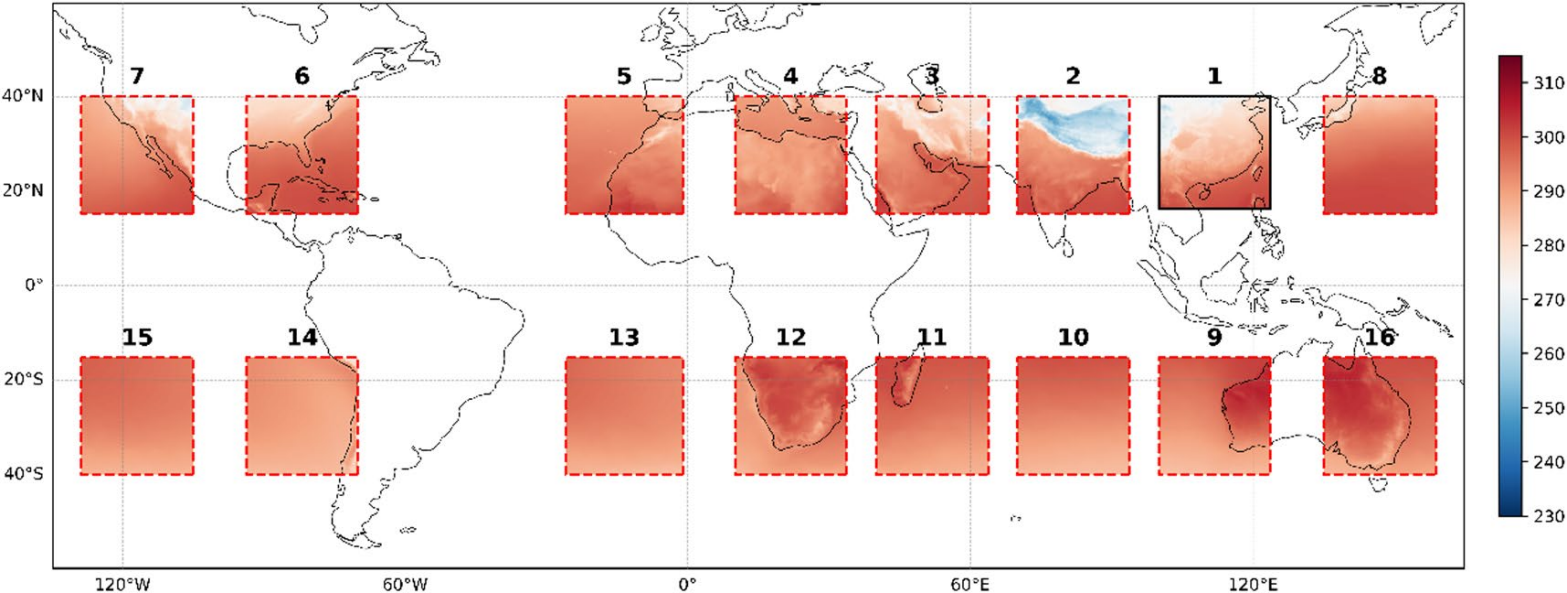
SR results for the test dataset average across 16 boxes at a resolution of 0.5° × 0.5°. The presented data is prepared by torch v.2.8.0 + cu126 https://pytorch.org/ and plotted by Matplotlib v.3.10.0 https://matplotlib.org/.

**Fig. 12:**
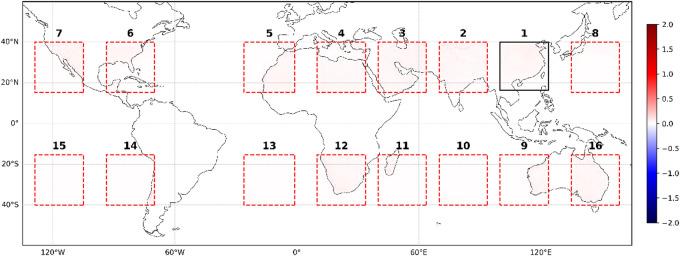
Comparison between real data and SR data for the test dataset average across 16 boxes at a resolution of 0.5° × 0.5°. The presented data is prepared by torch v.2.8.0 + cu126 https://pytorch.org/ and plotted by Matplotlib v.3.10.0 https://matplotlib.org/.

Figures 11 and 12 illustrated the SR for the test dataset average (from 1 January 2020 to 31 December 2020), showing that the model’s transfer learning works well over long-term periods. Like ResNet-Subpixel, the SR data from each box aligns closely with the real data over time. This is because the model averages out small - scale errors that occur at individual moments, enhancing overall performance.

With 4× downsampling, the SR performance of the model on the LR data is acceptable, and it completed transfer learning. Compared to 2× SR, the performance of 4× SR is somewhat reduced. The need to reconstruct a large amount of data leads to the loss of image detail information. Table 4 summarizes the performance of different downsampling rates for the test dataset average in each box.

**Table 4:** The SR performance of 2x and 4x SR for the test dataset average across 16 boxes.

Box	RMSE	MAE	MAPE	CORR	PSNR	SSIM
Box1-2×	0.2866	0.1774	0.063	0.9995	58.7328	0.9789
Box1-4×	0.5286	0.3498	0.1242	0.9989	53.5304	0.9589
Box2-2×	0.4836	0.2716	0.1097	0.9987	54.9524	0.9522
Box2-4×	0.7113	0.5253	0.1716	0.9981	51.0893	0.9188
Box3-2×	0.3852	0.2013	0.0893	0.9981	55.855	0.9643
Box3-4×	0.6401	0.4322	0.1447	0.9965	51.0064	0.9274
Box4-2×	0.3144	0.1285	0.068	0.9991	58.1811	0.981
Box4-4×	0.5728	0.3958	0.1321	0.9943	52.0117	0.9401
Box5-2×	0.3199	0.1783	0.0711	0.9993	58.0305	0.9818
Box5-4×	0.5591	0.4071	0.1109	0.9969	53.181	0.9369
Box6-2×	0.2886	0.1756	0.0595	0.9996	58.9248	0.9794
Box6-4×	0.4978	0.3185	0.1341	0.9991	54.1789	0.9577
Box7-2×	0.3338	0.1876	0.0697	0.9991	57.1439	0.9785
Box7-4×	0.6318	0.4099	0.1798	0.9957	52.6706	0.9416
Box8-2×	0.2294	0.1572	0.0617	0.9997	59.711	0.9712
Box8-4×	0.2781	0.1693	0.0591	0.9995	58.8743	0.9751
Box9-2×	0.3273	0.1521	0.075	0.9991	57.0971	0.9626
Box9-4×	0.3988	0.1822	0.0713	0.9989	52.4009	0.9591
Box10-2×	0.1361	0.1431	0.0418	0.9998	63.0185	0.9801
Box10-4×	0.2815	0.1731	0.0663	0.9995	58.7016	0.9773
Box11-2×	0.2198	0.1508	0.0491	0.9997	59.0813	0.981
Box11-4×	0.3841	0.1736	0.0751	0.9991	53.1671	0.9381
Box12-2×	0.3481	0.1861	0.0752	0.9987	56.4829	0.9608
Box12-4×	0.5476	0.3482	0.1131	0.9959	53.9188	0.9317
Box13-2×	0.1587	0.1482	0.0461	0.9998	62.4711	0.9877
Box13-4×	0.2598	0.1584	0.0609	0.9994	59.1822	0.9801
Box14-2×	0.2201	0.1558	0.0624	0.9996	58.7714	0.9718
Box14-4×	0.4018	0.231	0.0898	0.9962	54.1094	0.9647
Box15-2×	0.1264	0.1264	0.0411	0.9998	64.3011	0.9881
Box15-4×	0.2472	0.1463	0.1225	0.9947	58.5182	0.9882
Box16-2×	0.3526	0.1901	0.0811	0.9987	56.1152	0.9619
Box16-4×	0.5192	0.3327	0.1391	0.9974	53.7616	0.9317

### Data variables transfer

It is acknowledged that within the same area, there is a certain correlation among different meteorological quantities^50–52^. Consequently, we attempted to use ResNet-Subpixel to perform transfer learning for the four meteorological data—MSLP, 10 m-U, 10 m-V, and 2 m-D —within Box1. The pre-trained model is employed to conduct SR on 4 datasets and evaluate the model’s performance. In addition, we will conduct experiments on the four meteorological data for Box15, which performed best in the 2 m-T experiments, and Box2, which performed worst. The aim is to investigate the outcomes of transfer learning in terms of location and variables.

**Fig. 13:**
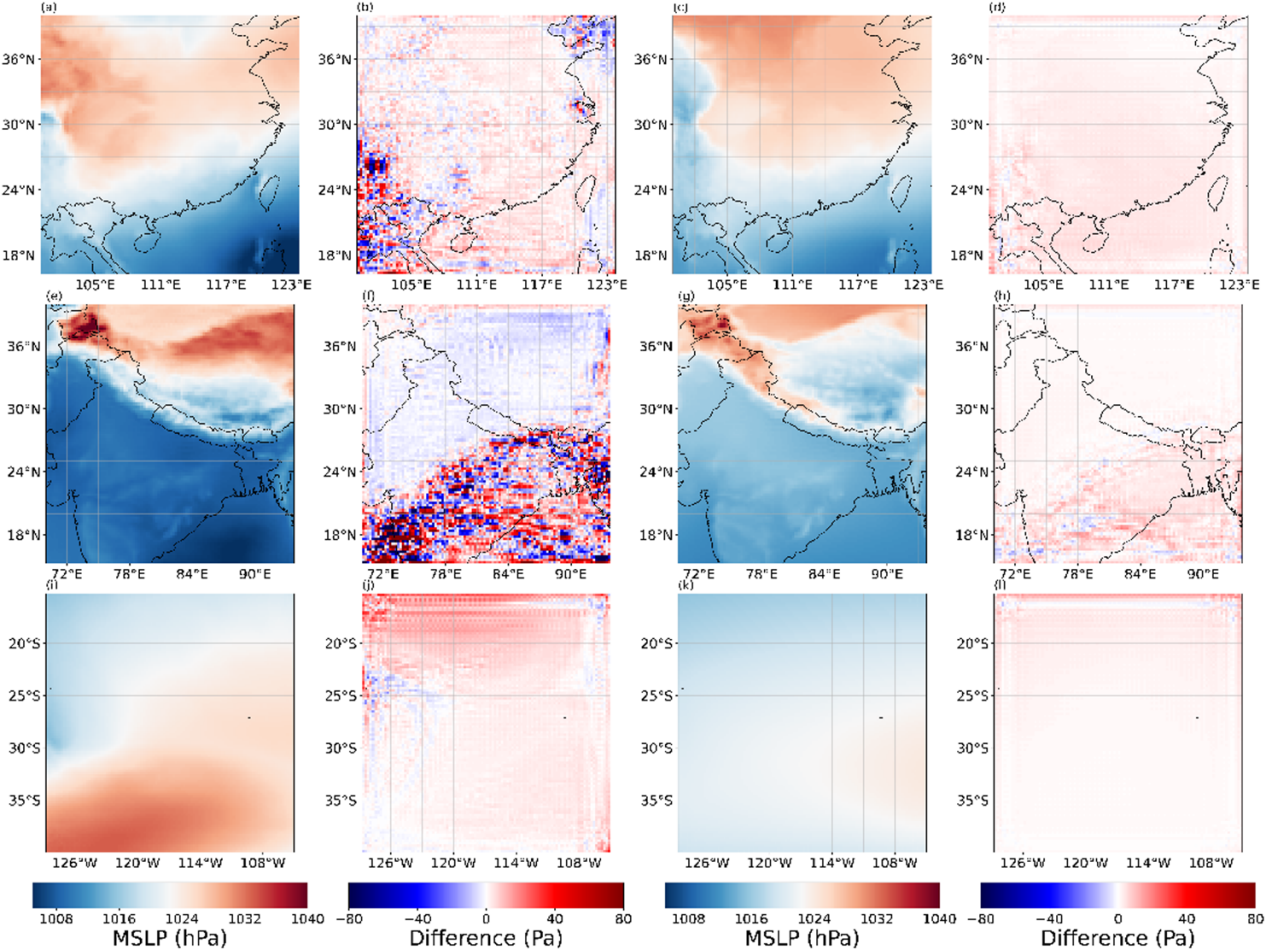
Evaluation of 2x SR performance for MLSP across regions of (a-d) Box1, (**e**–**h**) Box2, (**i**–**l**) and Box15. Columns represent: (**a**, **e**, **i**) performance at 2300 UTC 31 December 2020, (**b**, **f**, **j**) and with corresponding deviations from real data, (**c**, **g**, **k**) performance for the test dataset average, (**d**, **h**, **l**) and with corresponding deviations from real data. The presented data is prepared by torch v.2.8.0 + cu126 https://pytorch.org/ and plotted by Matplotlib v.3.10.0 https://matplotlib.org/.

**Fig. 14:**
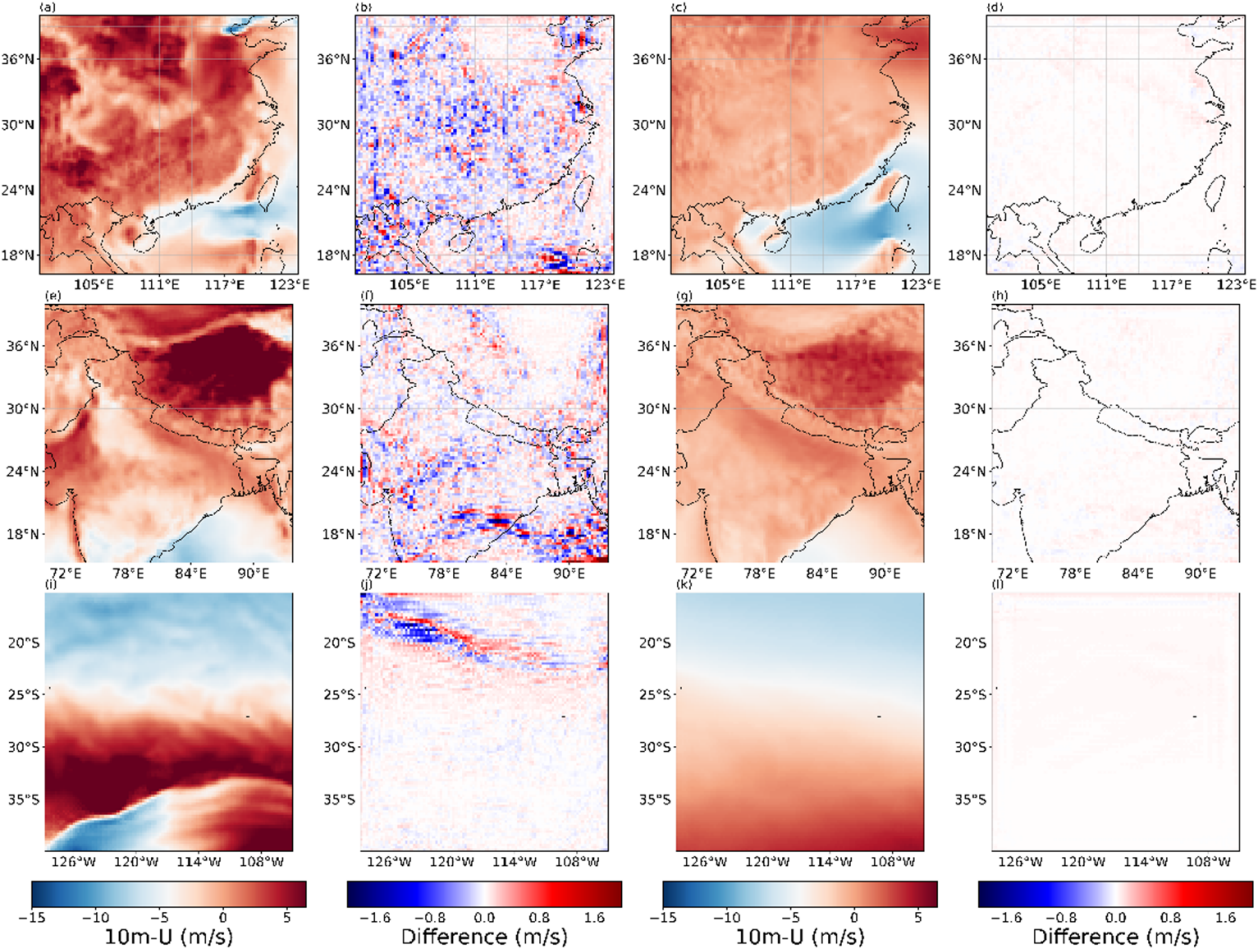
Evaluation of 2x SR performance for 10 m-U across regions of (**a**–**d**) Box1, (**e**–**h**) Box2, (**i**–**l**) and Box15. Columns represent: (**a**, **e**, **i**) performance at 2300 UTC 31 December 2020, (**b**, **f**, **j**) and with corresponding deviations from real data, (**c**, **g**, **k**) performance for the test dataset average, (**d**, **h**, **l**) and with corresponding deviations from real data. The presented data is prepared by torch v.2.8.0 + cu126 https://pytorch.org/ and plotted by Matplotlib v.3.10.0 https://matplotlib.org/.

**Fig. 15:**
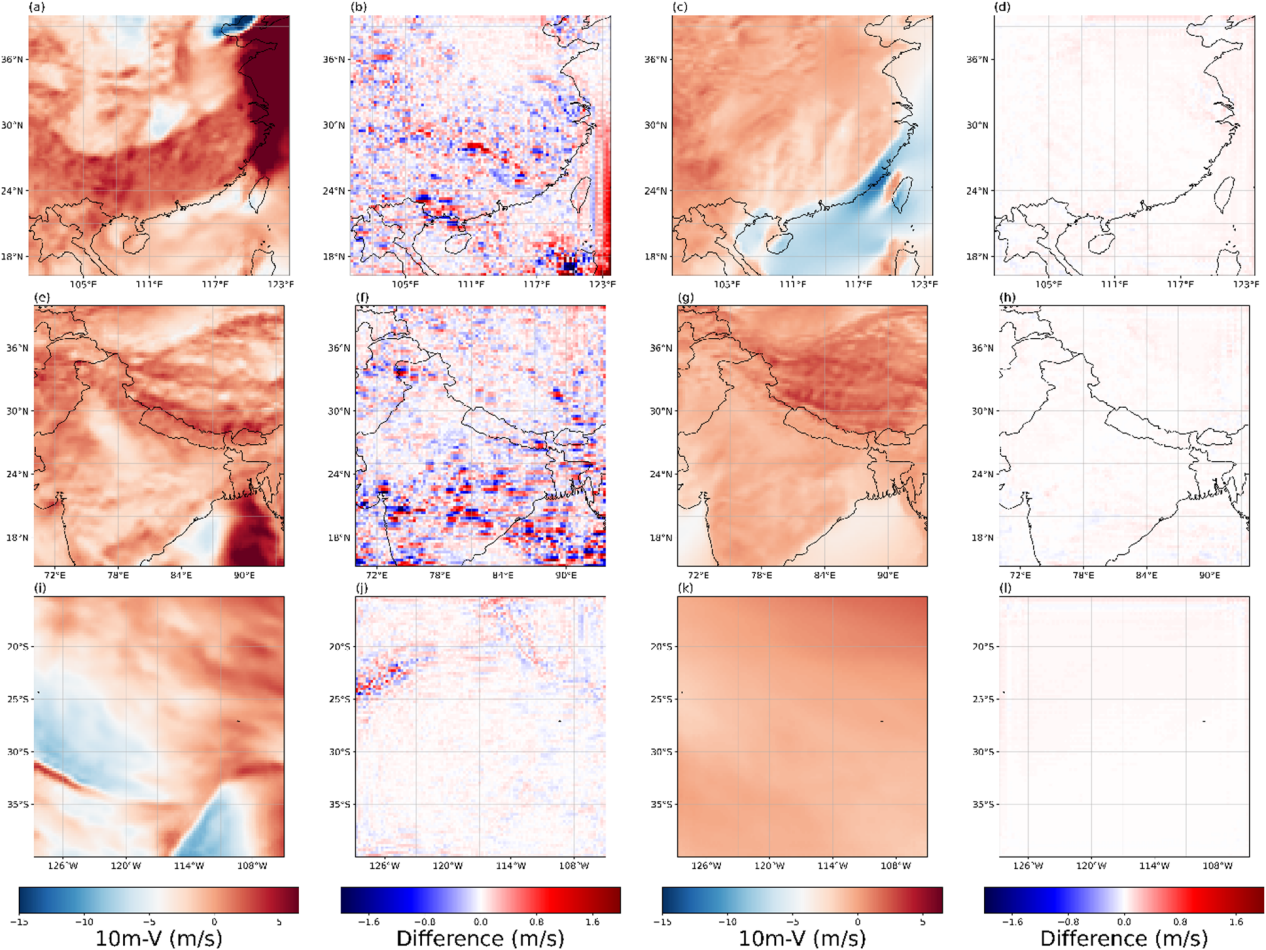
Evaluation of 2x SR performance for 10 m-V across regions of (**a**–**d**) Box1, (**e**–**h**) Box2, (**i**–**l**) and Box15. Columns represent: (**a**, **e**, **i**) performance at 2300 UTC 31 December 2020, (**b**, **f**, **j**) and with corresponding deviations from real data, (**c**, **g**, **k**) performance for the test dataset average, (**d**, **h**, **l**) and with corresponding deviations from real data. The presented data is prepared by torch v.2.8.0 + cu126 https://pytorch.org/ and plotted by Matplotlib v.3.10.0 https://matplotlib.org/.

**Fig. 16:**
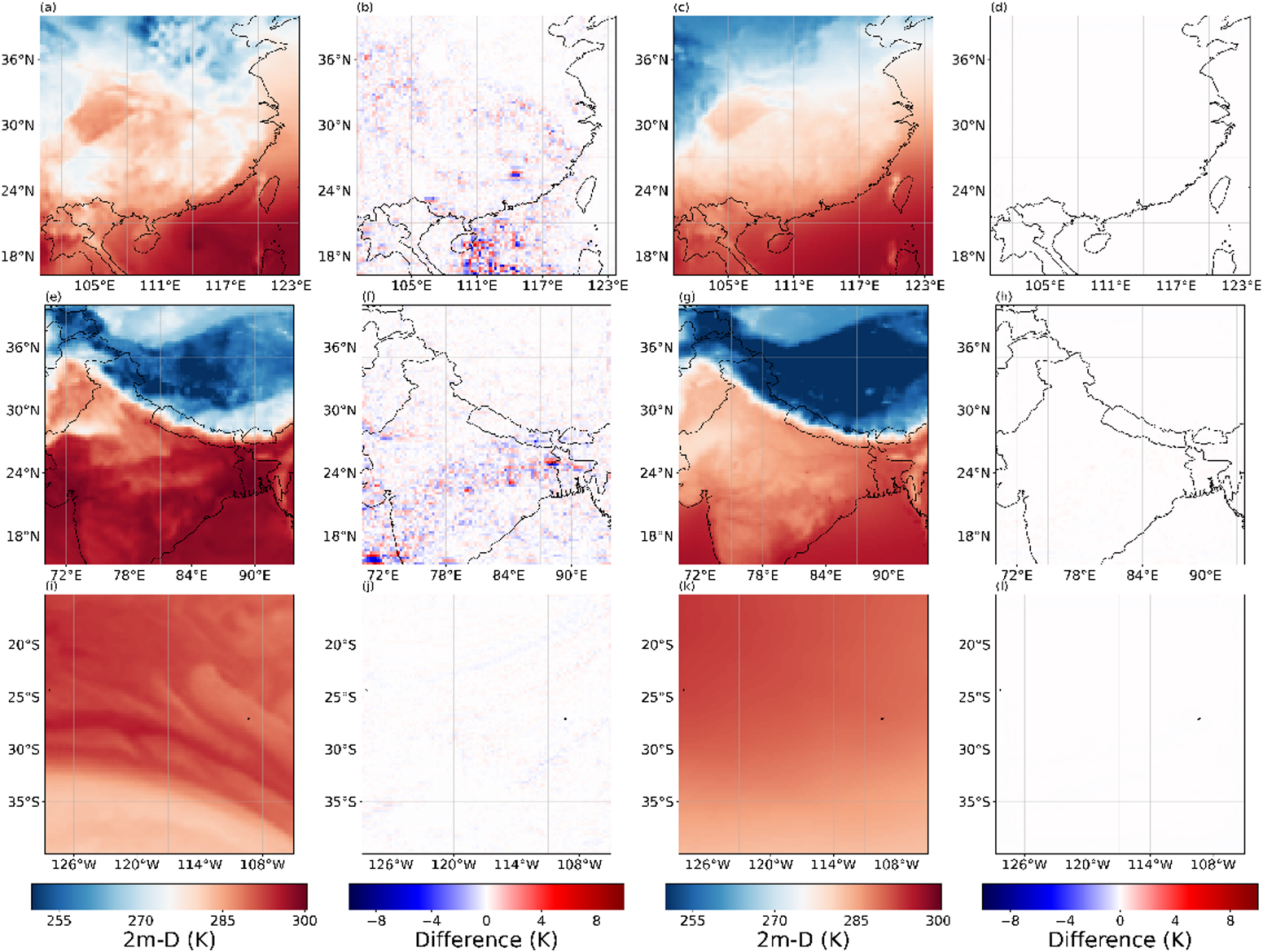
Evaluation of 2x SR performance for 2 m-D across regions of Box1, (**e**–**h**) Box2, (**i**–**l**) and Box15. Columns represent: (**a**, **e**, **i**) performance at 2300 UTC 31 December 2020, (**b**, **f**, **j**) and with corresponding deviations from real data, (**c**, **g**, **k**) performance for the test dataset average, (**d**, **h**, **l**) and with corresponding deviations from real data. The presented data is prepared by torch v.2.8.0 + cu126 https://pytorch.org/ and plotted by Matplotlib v.3.10.0 https://matplotlib.org/.

Figures 13, 14, 15 and 16 present the experimental results of four variables. Each row in the figures represents the regions of Box1, Box2, and Box15, respectively. Each column corresponds to the 2×SR results for the single moment, the differences between 2× SR data and real data for the single moment, the 2× SR results for the test dataset average, and the differences between 2× SR data and real data for the test dataset average. Comparison reveals that V-performed best overall (Fig. 15), followed by 10 m-U (Fig. 14), 2 m-D in third (Fig. 16), with MSLP (Fig. 13) is the worst. Consistently, oceanic regions outperformed land across all boxes. Notably, MSLP exhibited a significant SR performance drop at coastal zones compared to other variables. Unlike temperature and wind, MSLP in oceanic regions is more variable. However, the model still effectively extracts features and delivers good SR performance.As shown in Box2, a pronounced interface is consistently observed; correspondingly, most of reconstruction errors are spatially clustered along this interface. It shows that meteorological variables in the near-surface layer are disproportionately modulated by complex topography and land–sea transitions, rendering their super-resolution mapping exceptionally challenging.

To further validate the transfer effectiveness of the proposed model across meteorological variables, we compared with conventional traditional methods. ResNet-Subpixel found that Lasso is the best-performing method in Box1 aside from the model itself^30^. Therefore, Lasso was also employed in comparison with other regions. We evaluated the performance of SR on five variables. The experimental results are shown in Table 5. Overall, ResNet-Subpixel consistently demonstrated performance improvements. However, it is important to note that MSLP is particularly sensitive to position in transfer learning, and certain regions may exhibit suboptimal results. For 2 m-D, ResNet-Subpixel likewise outperforms conventional approaches over most regions; however, over spatially homogeneous and thermodynamically stable oceanic surfaces the super-resolution of humidity fields is inherently uncomplicated, permitting LASSO to deliver equally satisfactory performance.

**Table 5:** Comparison of the SR performance between ResNet-Subpixel and Lasso.

Box	Method	variable	RMSE	PSNR	SSIM
box1	ResNet-Subpixel	2 m-T	0.2897	58.8929	0.9721
		MSLP	9.4113	60.74516	0.9589
		10 m-U	0.2752	58.9837	0.9745
		10 m-V	0.3071	58.1851	0.9686
		2 m-D	0.3407	57.4645	0.9587
	Lasso	2 m-T	0.5426	53.1317	0.9465
		MSLP	13.1944	57.8109	0.9351
		10 m-U	0.3308	57.4893	0.9698
		10 m-V	0.3541	57.6544	0.9497
		2 m-D	0.4261	55.5421	0.9517
box2	ResNet-Subpixel	2 m-T	0.5298	53.6478	0.9481
		MSLP	25.2901	52.1597	0.9072
		10 m-U	0.2984	58.2074	0.9694
		10 m-V	0.3231	57.6416	0.9683
		2 m-D	0.5841	52.8026	0.9369
	Lasso	2 m-T	0.5743	53.3667	0.9481
		MSLP	20.8329	53.8437	0.9013
		10 m-U	0.3623	57.0019	0.9618
		10 m-V	0.4255	55.8472	0.9482
		2 m-D	0.5534	53.8772	0.9489
Box15	ResNet-Subpixel	2 m-T	0.2272	58.8718	0.9743
		MSLP	6.1723	64.4097	0.9783
		10 m-U	0.1547	64.1432	0.9807
		10 m-V	0.1745	61.2974	0.9802
		2 m-D	0.1626	62.326	0.9836
	Lasso	2 m-T	0.1646	60.7241	0.9813
		MSLP	8.1275	62.0192	0.9639
		10 m-U	0.2018	62.0532	0.9754
		10 m-V	0.2273	61.0325	0.9688
		2 m-D	0.1594	62.8255	0.9875

### Extreme weather transfer

Extreme weather events remain as a command of sustained attention in meteorological research. To evaluate model transfer performance under such extreme conditions, we perform SR experiments over specific regions during severe weather episodes. Among various types of extreme meteorological phenomena, hurricanes are chosen as a representative case due to their high annual frequency and significant socio-economic impacts. Consequently, assessing model transferability in hurricane scenarios holds substantial practical relevance^53^. We selected the Pacific (Boxes 1 and 7) and Indian Ocean (Box 2) regions. For each region, we assembled meteorological data for five tropical cyclones events that affected each of these regions and subsequently performed SR reconstruction on the variables, including 2 m-T, MSLP, 10 m-U, 10 m-V, and 2 m-D.

**Fig. 17:**
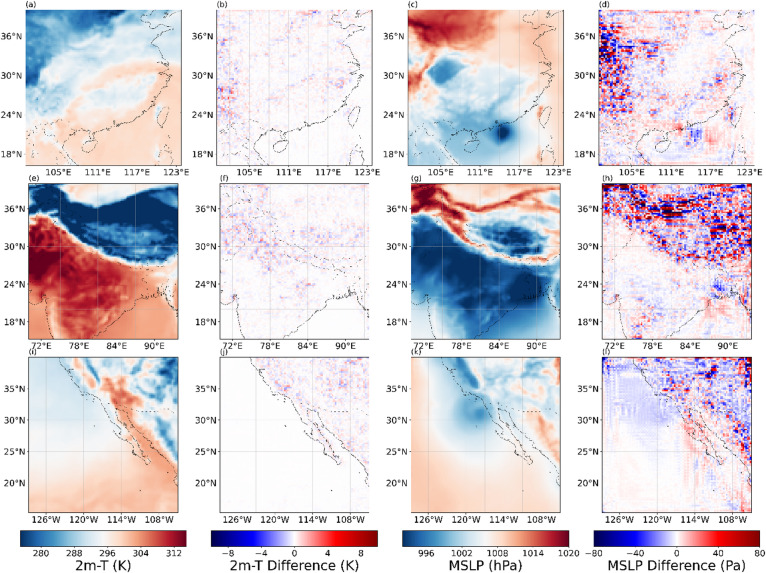
Evaluation of 2x SR performance under extreme-climate conditions of (**a**–**d**) Box1, (**e**–**h**) Box2, (**i**–**l**) and Box7. Columns represent: (**a**, **e**, **i**) SR results for 2 m-T, (**b**, **f**, **j**) and with corresponding deviations from real data, (**c**, **g**, **k**) SR results for MSLP, (d, h, l) and with corresponding deviations from real data. The presented data is prepared by torch v.2.8.0 + cu126 https://pytorch.org/ and plotted by Matplotlib v.3.10.0 https://matplotlib.org/.

Table 6. quantifies the average performance of each meteorological variable across the three regions during five tropical-cyclone events and further documents the average magnitude of model degradation under extreme-weather conditions. The magnitude of degradation as

9 $$\begin{array}{*{20}{c}} {Relative=({V_{Extreme}} - {V_{Normal}})/{V_{Normal}}} \end{array}$$

where, $\:Relative$ denotes the degradation magnitude of model performance; $\:{V}_{Normal}$ is the metric under normal conditions, and $\:{V}_{Extreme}$ is the corresponding value under extreme weather. Notably, a positive Relative for RMSE indicates performance degradation, whereas for PSNR and SSIM, a negative Relative value signifies deterioration. Evidently, Box2 yields the lowest scores, with MSLP and 2 m-D exhibiting pronounced degradation relative to the pre-training region. This deterioration is attributable to the fact that topographic complexity exerts a strong^54^, spatially heterogeneous forcing on both surface pressure and moisture tendency fields, thereby severely constraining the efficacy of super-resolution reconstruction. We contend that the model’s transferability is significantly constrained under conditions of complex land–sea topographic variability. Although a certain amount of HR data can still be retrieved, attaining more accurate representations necessitates explicit accounting for topographic heterogeneity. Figure 17. illustrates SR results for 2 m-T and MSLP under extreme weather conditions for the single moment.

**Table 6:** The SR performance of 2x SR for each meteorological variable under extreme weather conditions on average across 3 boxes.

Variables	RMSE	Relative (%)	PSNR	Relative (%)	SSIM	Relative (%)
Box1-2 m-T	0.3049	6.39	58.2914	−1.03	0.9732	−0.63
Box1-MSLP	13.1577	11.66	57.9830	−1.01	0.9393	−0.41
Box1-10 m-U	0.3054	13.20	58.2336	−0.96	0.9671	−1.06
Box1-10 m-V	0.3124	9.58	57.8782	−1.37	0.9673	−0.90
Box1-2 m-D	0.3377	8.28	57.3674	−1.52	0.9596	−0.62
Box2-2 m-T	0.5319	11.24	53.6647	−2.53	0.9490	−0.35
Box2-MSLP	24.3704	7.41	52.1914	−0.22	0.9088	−0.47
Box2-10 m-U	0.3098	15.97	57.7458	−1.69	0.9687	−0.97
Box2-10 m-V	0.3327	4.89	57.2089	−1.16	0.9593	−0.73
Box2-2 m-D	0.6423	9.97	51.2693	−2.90	0.9165	−2.17
Box7-2 m-T	0.3509	5.12	57.1103	−0.06	0.9744	−0.42
Box7-MSLP	13.1833	11.90	57.5209	−1.33	0.9445	−0.91
Box7-10 m-U	0.3267	13.87	57.2882	−0.80	0.9602	−0.52
Box7-10 m-V	0.3276	11.22	57.1905	−1.03	0.9596	−0.81
Box7-2 m-D	0.4070	9.36	56.2061	−0.67	0.9442	−0.59

## Discussion

We conducted multiple rounds of experiments to validate the transfer learning of the model. In regional transfer, model performed well; however, the results also revealed that topographic factors exert a significant influence on performance. Specifically, over complex land areas the performance generally declined—the worst box (Box2) dropped by approximately 6.88% in PSNR, whereas climatically stable oceanic regions achieved superior accuracy, with the best box (Box15) improving by 8.65%.

In data variables transfer, the model likewise exhibited robust performance. Notably, U-wind and V-wind attained accuracies comparable to those of the pre-trained variables. By contrast, MLSP and 2 m-D displayed pronounced fluctuations; constrained by multiple confounding factors, model skill for these fields remained limited. To evaluate the model under extreme weather conditions, we conducted tropical cyclones events experiments across different regions. Although SR performance for atmospheric variables declined within each box, the magnitude of degradation remained acceptable.

## Conclusions

This paper investigates the transfer learning capabilities of the ResNet-Subpixel model in meteorological data, encompassing the transfer of regional temperature data and the transfer among different variables within the same region. Through multiple experimental setups, the results demonstrate that the transfer learning model achieves satisfactory performance across various metrics. The model is capable of effectively transferring the feature information learned from the pre-training data to new regions or variables. Even when applied to common atmospheric variables such as MSLP, 10 m-U, 10 m-V, and 2 m-D, it maintains robust performance. Compared with traditional methods, the transferred model exhibits superior performance. This further underscores the potential application of transfer learning in the field of meteorology. Effective transfer learning not only reduces the demand for large data volumes in research but also shortens the time required for model training.

Additionally, we observed that the effectiveness of transfer learning is significantly influenced by geographical location, with more pronounced effects in oceanic regions compared to land areas. We attribute this to the complex topography of land, which leads to more variable climatic conditions. In future research, we will consider incorporating topographical features and other relevant information to assist model learning, thereby enhancing the accuracy of SR reconstruction.

## Data Availability

The datasets analyzed during the current study are available in the European Centre for Medium-Range Weather Forecasts (ECMWF) Re-Analysis v5 repository, [https://cds.climate.copernicus.eu/datasets/reanalysis-era5-single-levels? tab=download].
